# AI-driven marketing tactics and consumer behavior: a bibliometric review and systematic literature review

**DOI:** 10.3389/frma.2026.1914129

**Published:** 2026-08-12

**Authors:** Swati Ahuja, Amit Dutt, Nancy Sahni, Jaskiran Kaur

**Affiliations:** 1 Mittal School of Business, Lovely Professional University, Phagwara, Punjab, India; 2 Division of Academic Affairs, Lovely Professional University, Phagwara, Punjab, India

**Keywords:** AI-driven marketing tactics, artificial intelligence, bibliometric, SPAR-4-SLR, systematic literature review, TCM-ADO

## Abstract

**Objective:**

This study synthesizes the literature on AI-driven marketing tactics and their influence on consumer behavior. It identifies dominant theories, contexts, methodological approaches, and key antecedents that explain how AI-driven marketing tactics shape consumers' cognitive, emotional, and behavioral responses. This study also provides intellectual structure, publication trends and future research directions within this field.

**Methods:**

This study reviewed 155 articles, retrieved from the Scopus database and analyzed using bibliometric analysis, SPAR-4-SLR, and the TCM-ADO framework. The review covers publications from 2016 to 2026 and examines theoretical, contextual, and methodological approaches as well as factors influencing consumer responses to AI-driven marketing tactics.

**Findings:**

The findings reveal a significant growth in research publications related to AI-driven marketing tactics over the past decade. The analysis identifies dominant theories, research contexts, and methodological approaches employed in the field. Furthermore, a five-category antecedent framework was proposed, including AI characteristics, consumer psychological and behavioral traits, consumer attitudes and responses, marketing and communication factors, and contextual and situational factors. The results highlight that AI-driven marketing tactics influence consumer decision-making through technological, psychological, marketing and contextual mechanisms.

**Conclusion:**

The research on AI and consumer behavior has been expanded; however, literature remains fragmented. Most of the prior studies have examined individual AI tactics, such as chatbots, personalized recommendations and targeted advertisements in isolation instead of as an integrated AI-driven marketing tactic. Moreover, earlier studies focused on factors such as technology adoption and purchase intention, while fewer studies examined cognitive, emotional and behavioral mechanisms through which diverse AI-driven marketing tactics influence consumer behavior.

**Implications:**

The study provides a structured synthesis of literature and highlights gaps for future research. It suggests the need for greater theoretical integration, cross-cultural investigations, and longitudinal studies examining consumer responses to AI-driven marketing tactics. The findings provide valuable insights for researchers to advance the field and for practitioners aiming to design effective AI-enabled marketing strategies that enhance consumer engagement and decision-making.

## 1 Introduction

Artificial intelligence (AI) technology has transformed various sectors of the economy, especially the field of marketing and consumer behavior (Ziakis and Vlachopoulou, 2023). In this fast-paced environment, consumer needs and preferences are rapidly evolving. Understanding consumer behavior nowadays is very challenging with conventional marketing strategies (Arenas-Gaitán et al., 2019). To resolve this issue, firms use AI techniques to analyze large volumes of data. It also provides real-time consumer insights, develops customized products and services by predicting consumer behavior (Patil et al., 2024). Unlike traditional marketing tactics that primarily relied on descriptive data, AI-driven marketing tactics such as personalized recommendations, chatbots, automated emails, content creation, targeted advertising, dynamic pricing, and sentiment analysis are widely used to analyze consumer data and provide predictive insights in real time. It also helps firms in understanding the consumer reactions toward the products to enhance customer engagement, strengthen loyalty and shape buying decisions (Ahmad and Mir, 2025). For instance, personalized recommendations assist the consumer by suggesting products based on their preferences and narrowing their choices. Similarly, brands create content to emotionally engage customers on social media platforms. It helps companies accurately predict consumer behavior that ultimately increases their revenue and gains a competitive edge in the marketplace (Kumar et al., 2016). A prominent example is Amazon, which emerged as a market leader that uses AI tactics for consumer segmentation and individual support (AWS (Amazon Personalize), 2021). Over the years, artificial intelligence and consumer behavior have expanded significantly. However, earlier studies explored consumer behavior from different perspectives such as consumer trust, emotional responses, consumer responses, consumer perceptions and brand evaluation (Huang et al., 2024). Although, research on AI and consumer behavior has expanded, but the literature remains fragmented. Most studies have examined individual AI tactics, such as chatbots, personalized recommendations, and targeted advertisements in isolation, rather than providing a comprehensive synthesis of AI-driven marketing tactics as an integrated marketing approach. Moreover, earlier studies have focused on technology adoption and purchase intention, while comparatively less attention has been given to the cognitive, emotional, and behavioral mechanisms through which diverse AI-driven marketing tactics influence consumer behavior across different marketing contexts.

Prior research conducted by Mariani and Borghi (2019) and Haleem et al. (2022) mainly focused on the retail sector, and the findings offer limited insights of the application of artificial intelligence across sectors. Mariani et al. (2022) explored the relationship between AI in marketing, consumer behavior, and psychology. Jain et al. (2023) analyzed consumers' decision-making processes through the TCM-ADO framework, and Bohara et al. (2025) investigated the role of AI, Generative Artificial Intelligence (GAI) on consumer behavior. However, earlier studies investigated AI as a broad phenomenon, emphasizing how people adopt it and accept it. The present study focuses on the intersection between AI-driven marketing tactics and consumer behavior. Unlike previous reviews, which mainly focused on AI adoption, generative AI and specific application areas, this article provides an integrated synthesis of AI-driven marketing tactics and consumer behavior by combining bibliometric and TCM-ADO framework. This study identifies the dominant theories, contexts, methodologies, antecedents, decisions and outcomes in the literature. It also proposes five-category antecedents framework that organizes the fragmented literature into broader conceptual categories. By integrating bibliometric with TCM-ADO frameworks, this review not only maps the intellectual structure but also provides a comprehensive theoretical synthesis of AI-driven marketing tactics and consumer behavior. Based on the limitations from above-mentioned literature, this study seeks to answer these questions:

RQ1: Who are the most influential authors, journals, and publications in this field and how has the field progressed over time?
RQ2: Which theories, research contexts, and methodologies have been used to examine AI-driven marketing tactics and consumer behavior?
RQ3: What key antecedents, behavioral mechanisms and outcomes have been investigated in this domain?
RQ4: What future research directions and opportunities can be identified for the research endeavors?

To address these questions, the paper is organized in the following manner. Section 2 discusses the conceptual framework, Section 3 outlines the methodology, Section 4 reports the findings, Section 5 presents the TCM-ADO synthesis of the literature, and Section 6 highlights the future research directions.

## 2 Theoretical foundations

The paper explores the intellectual structure and theoretical foundations of AI-enabled marketing tactics in relation to consumer behavior, which provides the conceptual basis for a systematic literature review (SLR).

### 2.1 AI-driven marketing tactics

AI refers to computer systems that are capable of analyzing information and making decisions with minimal human interference. It has transformed marketing practices and reshaped the relationship between companies and customers (Sterne, 2017; Bag et al., 2022; Haleem et al., 2022).

AI-driven marketing tactics use algorithms to understand, forecast and shape consumer behavior by analyzing large volumes of consumer data, including past purchase behavior, browsing history, and social media interaction (De Bruyn et al., 2020). These tactics assist companies in reducing the acquisition costs and designing strategies to convert potential customers into actual consumers (Davenport et al., 2020). Companies use AI to provide personalized recommendations based on customers' behavior, anticipate future actions through predictive analytics, and alter the content in real-time. Programmatic advertising also uses algorithms to target customers and display advertisements according to the customer preferences (Malthouse et al., 2019). Moreover, automated email campaigns and sentiment analysis allow companies to send personalized content at optimal time in order to improve customer engagement (Santos et al., 2023). In addition, dynamic pricing enables organizations to offer exclusive discounts based on market situation and consumer preferences (Kopalle et al., 2023). Unlike conventional digital marketing, which generally delivers mass communications, AI-driven marketing tactics analyze large volume of consumer data and adapt marketing communication in real-time (Senyapar, 2024). This enables firms to deliver personalized recommendations, automated e-mails and targeted advertisements based on individual consumer preferences.

### 2.2 Consumer behavior

Consumer behavior refers to how people, groups, and businesses identify, purchase, utilize and discard the products, services and experiences to satisfy their needs and desires (Ma and Wang, 2024). Traditionally, theories focused on the rational decision-making of the consumer; whereas contemporary theories rely on social, cultural, psychological and personal factors that affect customer choice. Afterwards, omnichannel retailing and e-commerce emerged. As a result, marketers increasingly use artificial intelligence (AI) and extensive behavioral datasets to get insights into consumer's mental processes (Verhoef et al., 2021). The increasing use of AI has shifted consumer decision-making from rational evaluation to personalized recommendations and emotional engagement. Consequently, consumer behavior is no longer shaped by individual preferences but by AI marketing tactics that influence pre-purchase processing, purchase evaluation and post-purchase decisions (Berg, 2026).

### 2.3 AI-driven marketing tactics and consumer behavior

The integration of AI marketing tactics and consumer behavior has reshaped how consumers evaluate information, analyze, and make purchase decisions. These tactics help businesses to analyze large volume of consumers data that was not possible with conventional techniques (Davenport et al., 2020). Personalized recommendations, chatbots, targeted advertising, automated emails, and dynamic pricing increasingly influence consumers' attitudes, purchase decisions, and post-purchase experiences (Puntoni et al., 2021).

Previous studies have shown that AI-driven marketing tactics positively influence customer engagement, purchase intention, brand loyalty, and customer satisfaction (Hossain, 2024). However, these tactics also raise concerns about the privacy, transparency, ethical use of consumer data, and algorithmic bias (Gao et al., 2023). These findings highlight that AI tactics bring opportunities as well as create new obstacles in consumer decision-making.

Existing studies have explained consumer behavior from multiple theoretical perspectives. Consumer decision-making and behavioral theories explained how consumers evaluate AI tactics as stimuli and make purchase decisions. Technology adoption theories highlighted consumers' acceptance of AI technologies based on factors such as perceived usefulness, ease of use, and behavioral intention. Social and communication theories highlighted the significance of society, anthropomorphism, and human-like interactions, whereas psychological theories focused on cognitive and emotional responses such as trust, perceived risk, motivation, and fear of missing out.

Despite these theoretical perspectives, no theoretical framework captured consumer behavior in AI-driven digital environments. Existing studies have applied these theories in isolation, which resulted fragmented theoretical explanations of consumer behavior. This highlights the need for a comprehensive synthesis that integrates theoretical, contextual, and behavioral mechanisms to provide a broader understanding of how AI-driven marketing tactics influence consumer behavior.

## 3 Methodology

The systematic literature review (SLR) approach was used to address the research questions as it is considered an appropriate method for evaluating the existing studies (Al-Zubidy and Carver, 2019). It helps in identifying the research gaps and future research directions (Paul and Feliciano-Cestero, 2021; Danese et al., 2018).

To structure the review, this study adopts the TCM-ADO framework. It was developed by Paul et al. (2017) and provides the classification of previous studies based on theories, contexts and methodologies. Moreover, Paul and Benito (2018) proposed the ADO framework, which offers insights into antecedents, decisions, and outcomes. The integration of these frameworks provides a detailed understanding of the domain (Batham et al., 2025; Shi and Shen, 2025; Majcher, 2025; Jaziri et al., 2025; Lim et al., 2021; Singh and Negi, 2025).

Bibliometric analysis provides insights of large bodies of literature by identifying publication trends, influential authors, journals, organizations, and documents within a specific field (Chiu and Ho, 2007). However, bibliometric analysis alone may not fully capture the depth and breadth of the literature. Therefore, this study adopts a hybrid approach by integrating bibliometric analysis with TCM-ADO framework to get the quantitative and qualitative synthesis. The bibliometric analysis identifies the intellectual structure and publication trends, whereas the TCM-ADO framework provides a structured synthesis of theories, contexts, methodology, antecedents, decisions and outcomes. These approaches provide a comprehensive understanding of AI-driven marketing tactics and consumer behavior, and identify future research directions.

The screening and eligibility assessment were conducted by the author using the predefined inclusion and exclusion criteria to ensure consistency. The selection process consisted of the title and abstract screening, followed by full-text assessment of the articles.

The study followed the inclusion and exclusion criteria proposed by Paul and Criado (2020). The inclusion criteria were used to select the reviewed articles: (a) Peer-reviewed articles. (b) Articles indexed in Scopus database. (c) Articles published in English. (d) Articles related to AI-driven marketing tactics and consumer behavior. The exclusion criteria involved (a) Conference papers, book chapters, and books. (b) Articles published in languages other than English. (c) Studies unrelated to the research domain. (d) Articles not in final publishing stage.

After applying the inclusion and exclusion criteria, the review proceeded to the following stages.

Stage1: Acquisition. Stage2: Organization. Stage3: Purification. Stage4: Evaluation. Stage 5: Reporting.

### 3.1 Selection criteria

The Scopus database was selected because it is considered one of the prominent databases and offers a rich body of literature (Fahimnia et al., 2015). It provides peer-reviewed literature, which is accepted in the global academic community (Pranckut, 2021) and offers consistency as different databases have their own standards (Zhu and Liu, 2020). In addition, Scopus database is regularly updated and can be integrated with other tools such as VOSviewer.

The search was conducted in a Scopus database under the Title, Abstract, and Keywords field using the following keywords ((“artificial intelligence marketing tactic^*^”) OR (“AI marketing tactic^*^”) OR (“AI marketing strateg^*^”) OR (“predictive analytics”) OR (“personali^*^ed recommendation^*^”) OR (“chatbot^*^”) OR (“AI dynamic pricing”) OR (“automated email” OR “automated e-mail”) OR (“targeted advertisement^*^” OR “targeted advertising”) OR (“sentiment analysis”)) AND (consumer behavio^*^r). The initial search returned 1,444 articles. The data was extracted in 2026 as shown in Table 1. To obtain relevant studies, the articles were screened using the predefined criteria. The following criteria were applied to filter the data:

1) The subject category of Business, Management, and Accounting and Economics, Econometrics and finance were selected, which reduced the number of articles to 516.
2) Only peer-reviewed journal articles were considered, reducing the count to 331 (after excluding conference papers, conference proceedings, book chapters, books, patents, and other non-peer-reviewed documents).
3) Articles published in English were retained, resulting of 328 articles.
4) Duplicate records were removed, and the remaining articles were screened based on their titles and abstracts, which resulted of 224 articles.
5) After that, there was full-text assessment for eligibility, the final sample consisted of 155 articles.

**Table 1:** SPAR-4-SLR framework for identifying and selecting the articles from Scopus database.

Section	Details
IDENTIFICATION	Domain: AI-driven marketing tactics and consumer behavior Research Questions: RQ1: Who are the most influential authors, journals, and publications in this field and how has the field progressed over time? RQ2: Which theories, research contexts, and methodologies have been used to examine AI-driven marketing tactics and consumer behavior? RQ3: What key antecedents, behavioral mechanisms, and outcomes have been investigated in this domain? RQ4: What future research directions and opportunities can be identified for the research endeavors? Source type: Articles Source Quality: Scopus
ACQUISITION	Database utilized: Scopus Timeframe: 2016–2026 Keywords searched: ((“artificial intelligence marketing tactic^*^”) OR (“AI marketing tactic^*^”) OR (“AI marketing strateg^*^”) OR (“predictive analytics”) OR (“personali^*^ed recommendation^*^”) OR (“chatbot^*^”) OR (“AI dynamic pricing”) OR (“automated email” OR “automated e-mail”) OR (“targeted advertisement^*^” OR “targeted advertising”) OR (“sentiment analysis”)) AND (consumer behavio^*^r). Initial Retrieval: 1,444 records
ORGANIZATION	Code: article title, keywords, journal, authors, theory, context, methodologies and study characteristics. Framework: TCM-ADO
PURIFICATION	Article type excluded: conference papers, conference proceedings, books, book chapters, articles published in languages other than English, articles not in the final publication stage, duplicate records and articles not relevant to AI-driven marketing tactics and consumer behavior. Final articles included: 155 journal articles.
EVALUATION	Analytical approach: bibliometric analysis with TCM-ADO framework Expected outcome: identification of publication trends, intellectual structure, dominant theories, research contexts, methodologies, antecedents, decisions, outcomes, and future research directions.
REPORTING	Reporting format: narrative discussion study limitation: non-empirical funding statement: no external finance assistance

## 4 Bibliometric characteristics of the studies

This section analyses the documents retrieved from the Scopus database to identify the publication trends, leading countries, journals, and citation impact. To enhance precision, this study was conducted using SLR with bibliometric analysis (Phulwani et al., 2020; Zupic and Cater, 2015).

Bibliometric analysis, also known as science mapping, is used to figure out the productivity and impact of research articles. Using statistical methods, publication trends were examined, such as prolific authors, countries, sources and institutions (Lim and Kumar, 2024; Nirmal et al., 2024; Sahoo, 2022). After conducting a bibliometric analysis, the retrieved Scopus dataset was synthesized using the TCM-ADO framework to identify dominant theories, contexts, methodologies, antecedents, decisions, outcomes, and future research directions.

### 4.1 Emerging publication trends

**Figure 1:**
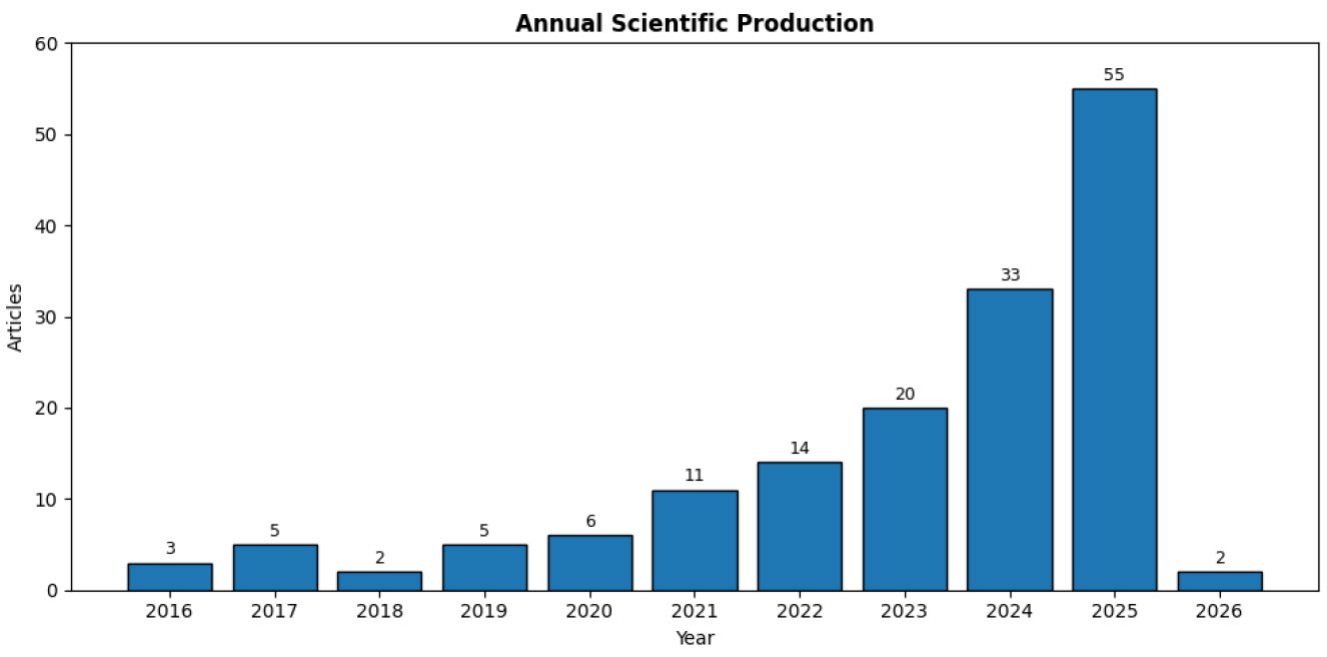
Publication trends from 2016 to 2026.

Figure 1 shows the growth in scholarly attention in this field over the decade. In the beginning years, from 2016 to 2020, the topic appeared to be in its infancy, with 3 and 5 articles in 2016 and 2017. The publication declined to 2 articles in 2018. In 2019 and 2020, publication output increased to five and six articles. From 2021, there has been a rise in research interest and contributions. A substantial proportion of publications accounted from 2021 to 2025 (78% of 155 articles). This growth may be attributed to advancements of artificial intelligence in marketing and increased digitalization (Hendrayati et al., 2024). Furthermore, COVID-19 may have accelerated the shift toward online platforms (Nanda et al., 2021) and increased scholarly interest in understanding psychological and behavioral implications for consumers, which can be examined through the TCM-ADO framework.

### 4.2 Distribution of publications by country

**Figure 2:**
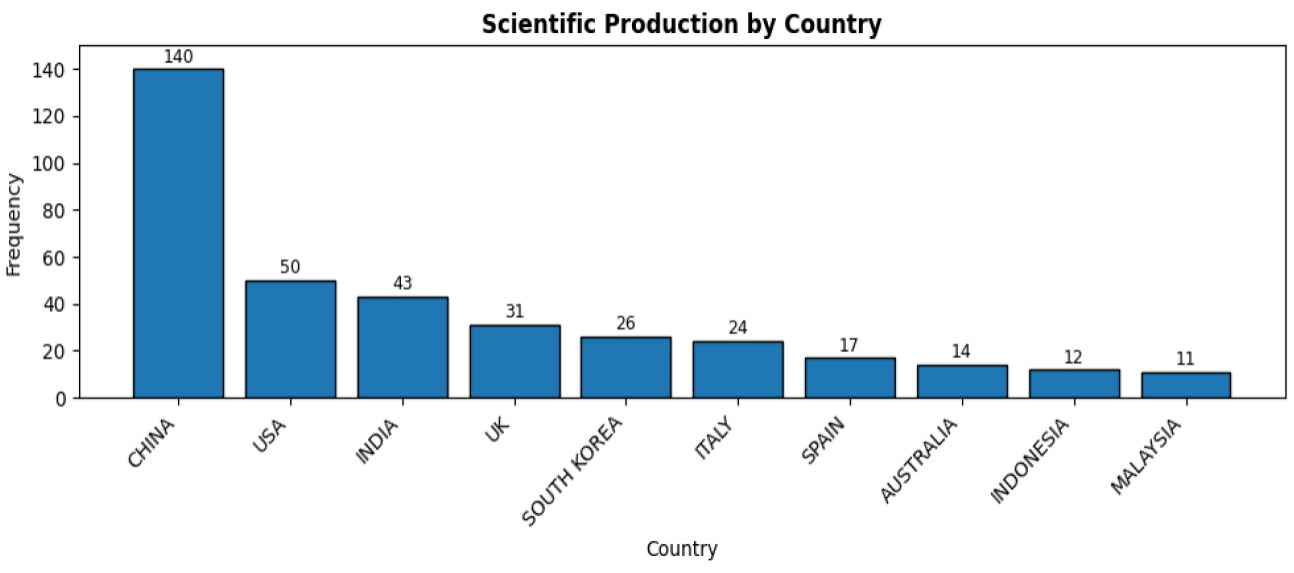
Top 10 countries with the highest contribution.

Figure 2 depicts research publications in this domain across countries. China, the USA, and India were the top three contributors. China emerged as a significant contributor, followed by the USA and India, which indicates growing global interest in AI-driven marketing and consumer behavior research. Along with these, South Korea and the United Kingdom also contributed significantly to this domain. The dominance of publications in particular geographical areas is associated with substantial investment in artificial intelligence, digital infrastructure and technology-driven marketing (Bazavan and Huidumac-Petrescu, 2022). Conversely, limited contribution in developing countries indicates opportunities for cross-cultural investigations to examine the consumer responses to AI-driven tactics used by the marketers.

### 4.3 Publication outlets

**Figure 3:**
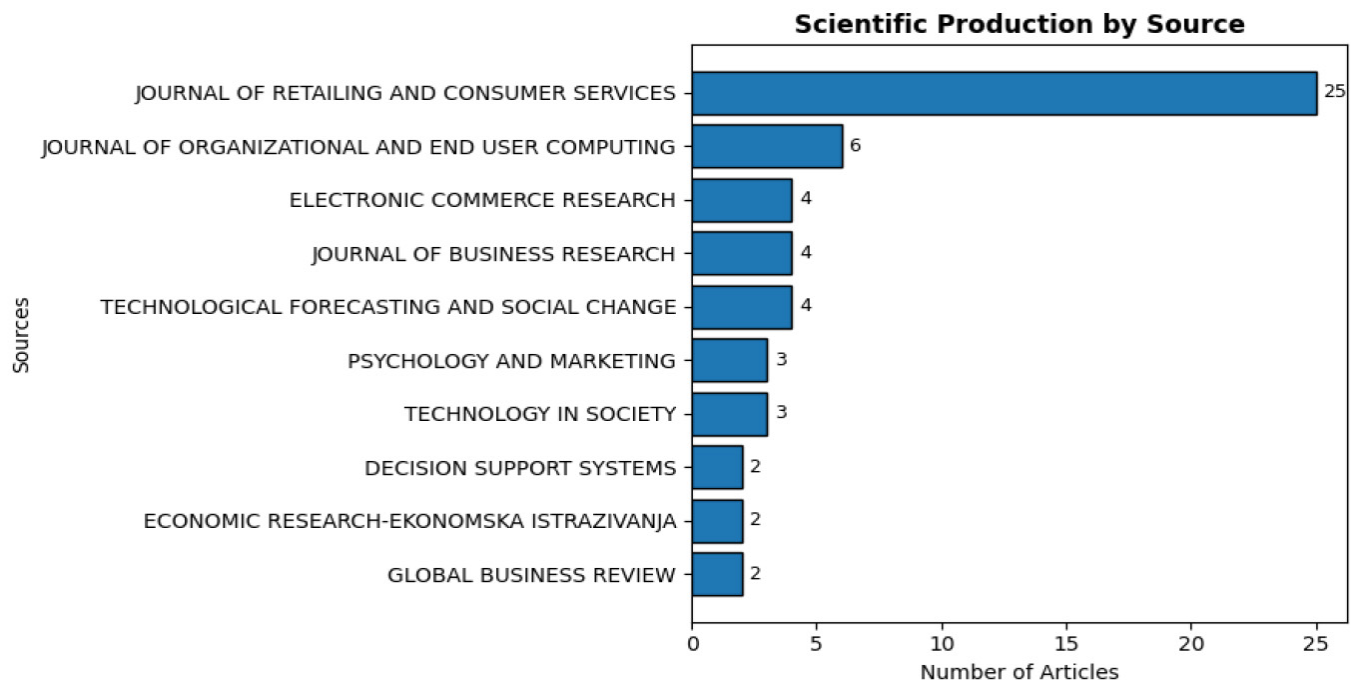
Most relevant Journals.

Figure 3 shows the journals that have contributed most to research in this field. The Journal of Retailing and Consumer Services is a prominent journal with 25 contributions, followed by the Journal of Organizational and End User Computing, which published six articles. Electronic Commerce Research, Journal of Business Research, and Technological Forecasting and Social Change each published four articles. Both Psychology and Marketing and Technology in Society contributed three articles each. Moreover, Decision Support Systems, Economic Research, and Global Business Review also published two articles related to the domain. The dominance of marketing and technology journals highlights that research has mainly focused on technological innovation and marketing applications. However, there were fewer publications in psychology and consumer behavior journals, which highlight that the cognitive and emotional mechanisms in respect AI-driven marketing remain underexplored.

### 4.4 Top cited publications

Table 2 summarizes the top 10 publications, each of which has received more than 100 citations and has had a substantial influence on the literature. The research by Liu and Mattila (2017) has the highest citation count of 264, which highlights a foundational study in this domain. Song et al. (2022) ranked second among the most cited articles in this research field. The highly cited publications primarily focused on adoption of chatbots, targeted advertisement, and personalized recommendation, indicating that these topics have formed foundation of research of the field. However, few studies have synthesized these individual AI-driven marketing tactics as an integrated framework. Moreover, the present study provides comprehensive synthesis of the literature and their influence on consumer's cognitive, emotional and behavioral responses.

**Table 2:** Most cited publications.

Author (year)	Article title	Total citations
Liu and Mattila (2017)	Airbnb: online targeted advertising, sense of power, and consumer decisions	264
Song et al. (2022)	Will artificial intelligence replace human customer service? The impact of communication quality and privacy risks on adoption intention	216
Villarroel Ordenes et al. (2017)	Unveiling what is written in the stars: analyzing explicit, implicit, and discourse patterns of sentiment in social media	206
Pantano and Pizzi (2020)	Forecasting artificial intelligence on online customer assistance: evidence from chatbot patents analysis	171
Jacobson et al. (2020)	Social media marketing: who is watching the watchers?	169
Pizzi et al. (2021)	Artificial intelligence and the new forms of interaction: who has the control when interacting with a chatbot?	162
Lalicic and Weismayer (2021)	Consumers' reasons and perceived value co-creation of using artificial intelligence-enabled travel service agents	156
Kim et al. (2017)	What makes tourists feel negatively about tourism destinations? Application of hybrid text mining methodology to smart destination management	154
Summers et al. (2016)	An audience of one: behaviorally targeted ads as implied social labels	130
Tran et al. (2021)	Exploring the impact of chatbots on consumer sentiment and expectations in retail	113

### 4.5 Most prominent authors

**Figure 4:**
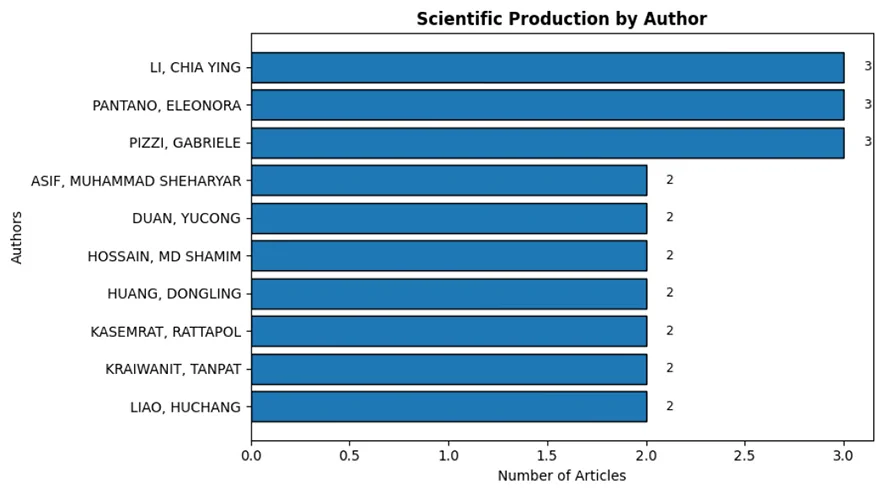
Most prominent authors.

**Figure 5:**
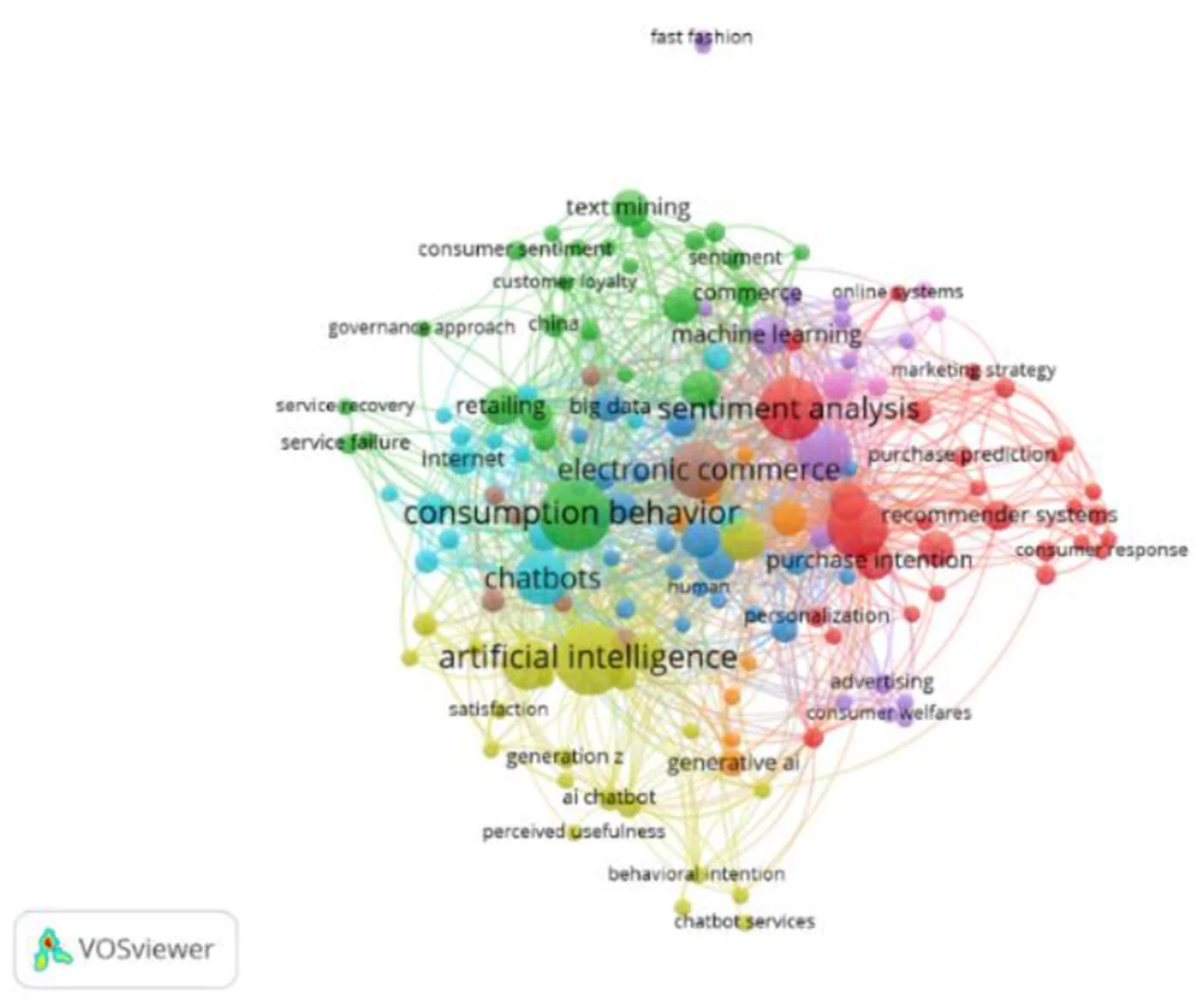
Co-occurrence keyword.

Figure 4 highlights the most prominent researchers in this domain. Li Chia Ying, Pantano Eleonora and Pizzi Gabriele have been identified as the most prolific authors, each with three publications. In addition, Asif Muhammad Sheharyar, Duan Yucong, Hossain Md Shamim, Huang Dongling, Kasemrat Rattapol, Kraiwani Tanpat, and Liao Huchang have contributed two articles each to this research area, and indicating that they are emerging contributors in this domain. Figure 5 displays the co-occurrence keyword analysis, which highlights major research themes and evolving trends.

### 4.6 Keyword analysis

The keyword analysis revealed that research has focused on consumption behavior, purchase intention, chatbots, sentiment analysis, retail and e-commerce. These themes are further synthesized with the help of the TCM-ADO framework, which provides theoretical foundations, research context, methodologies, antecedents, decisions, and outcomes. Thus, the bibliometric analysis provides the intellectual structure and the systematic literature review offers a deeper conceptual interpretation of the identified research themes through the TCM-ADO framework.

The bibliometric analysis highlights various dominant research clusters centered on consumer behavior. However, it fails to explain the underlying theoretical, contextual, and behavioral relationships among these themes. Therefore, the subsequent TCM-ADO framework extends the bibliometric findings and synthesizes the dominant outcomes associated with this research. In this way, the bibliometric analysis identifies what has been studied, whereas the systematic literature review explains how and why these themes have evolved.

## 5 General overview: TCM-ADO framework

This framework is used to organize, synthesize and categorize the literature. It systematically presents the key components, that is, theories, contexts, methods, antecedents, decisions, and outcomes related to AI-driven marketing tactics and consumer behavior (Paul et al., 2021). This framework provides the insights of theories applied and developed in this field. The contextual framework highlights various situations and conditions used in the prior studies. Moreover, the methodology section provides an overview of the techniques used to illustrate AI-driven marketing tactics and consumer behavior (Lim et al., 2021). Lastly, key antecedents, behavioral mechanisms and outcomes provide a comprehensive overview of the constructs and variables used in the previous studies. The integration of both bibliometric analysis and TCM-ADO framework indicates quantitative mapping and qualitative synthesis of the theoretical, contextual, methodological and behavioral dimensions.

### 5.1 Theories

Theories provide a theoretical and conceptual framework that guides scholars to meet their research objectives. Basically, theories provide the foundation on which results of the studies are based (Lim et al., 2021).

The theoretical analysis (Table 3) highlights that research is basically grounded on consumer decision-making and behavioral theories, followed by social and communication theories, technology adoption frameworks, media and interaction theories, and motivational and psychological theories. These theories indicate that scholars investigated consumer responses from multiple perspectives, such as rational decision-making, social influence, technology acceptance, user experience, and psychological motivation.

**Table 3:** Theories employed in this domain.

Theories	No. of articles
Technology adoption and acceptance technology	
Acceptance model/extended TAM	5
Expectation confirmation model	1
UTAUT and UTAUT2	2
Service robot acceptance model	1
DeLone and McLean IS success model	1
Extended AIDUA model	1
Consumer decision-making and behavioral theories	
Customer satisfaction theory	1
Status quo bias theory	1
Rational choice theory	1
Multi-attribute utility theory (MAUT)	1
Theory of reasoned action	1
Planned behavior theory	5
Behavioral reasoning theory	1
Regret theory	1
Value-attitude-behavior model	1
Expectancy-value theory	1
Perceived value theory	1
Value theory	1
Attribution theory	1
Perceived value theory	1
Value theory	1
Attribution theory	1
Psychological accounting theory	1
Social exchange theory	3
Resource exchange theory	1
Nudge theory	1
Motivation, emotion, and psychological	
Self-determination theory	2
Psychological reactance theory	1
Self-perception theory	1
Emotional intensity theory	1
Implicit personality theory	1
Behavioral plasticity theory	1
Psychological theory of fun	1
Social Cognitive theory	1
Stressor-strain-outcome (SSO)	1
Value–belief–norm (VBN) theory	1
Optimal stimulation level theory	1
Affordance and actualization theory	3
Self-congruence theory	1
Social and communication theories	
Social support theory	1
Social identity theory	4
Social comparison theory	1
Social presence theory	2
Social and communication theories	
Social support theory	1
Social identity theory	4
Social comparison theory	1
Social presence theory	2
Communication privacy management theory	1
Social response theory	1
Anthropomorphism theory	3
Mind perception theory	2
Information spillover theory	2
Role congruity theory	1
Information theory	1
Persuasion theory	1
Media, interaction, and user experience theories	
Uses and gratifications theory	3
Stimulus-Organism-Response (SOR) model	2
Speech and act theory	1
Assemblage theory	1
Humanizing experience theory	1
Cultural convergence theory	1
Cybernetics theory	1
Expectancy violations theory	1

The dominance of consumer decision-making and behavioral theories indicates that previous studies have mainly focused on how consumers analyze information and make decisions. A large proportion of studies employed choice-based theories, including Rational Choice Theory, Multi-Attribute Utility Theory, Expectancy Value Theory, Customer Satisfaction Theory, and Social Exchange Theory. These frameworks consider that consumers evaluate information logically and make decisions based on benefits and costs. These theories provide valuable insights of consumer experience in response to the AI-driven marketing tactics, which do not fully capture the complexity in the digital environment. AI-driven marketing tactics such as algorithmic recommendations, automated emails, and targeted advertisements influence consumer reactions beyond rational consideration as their responses are shaped by emotional, cognitive, and social mechanisms (Kim, 2025).

The growing use of social and communication theories reflects the significance of society in the AI digital environment. The application of Social Identity Theory, Social Comparison Theory, and Anthropomorphism Theory indicates that consumer responses are influenced by a sense of belonging, peer comparisons, and human-like interactions. In particular, the emergence of anthropomorphic, such as chatbots and virtual assistants, reflects a shift from transactional exchanges toward socially embedded interactions, in which trust, emotional attachment, and perceived humanness influence consumer experiences. However, existing research provides a limited understanding of how AI-driven marketing tactics intensify social comparison and shape behavioral outcomes.

The increasing application of media, interaction and user experience theories highlights how consumer behavior is shaped by interactive experiences rather than passive exposure of brand messages. The widespread adoption of the Stimulus–Organism–Response (SOR) framework shows that AI-driven marketing tactics serve as stimuli that influence cognitive and emotional states, which lead to affective behavioral outcomes (Zhao et al., 2025; Haneefa and Singh, 2025). Moreover, theories such as Uses and Gratifications Theory, Expectancy Violations Theory, and Assemblage Theory highlight the experiential nature of digital interactions.

From a technology adoption perspective, TAM has been widely used to explain consumer engagement with AI marketing tactics. Existing studies largely focused on perceived usefulness, ease of use, credibility, and functionality as key elements of acceptance. On the contrary, less attention has been given to ethical concerns, emotional responses, privacy perceptions, and algorithmic transparency, despite their increasing relevance in AI-driven marketing tactics. Moreover, 74 of the prior studies did not employ any explicit theoretical framework, which indicates that a substantial proportion of the literature remains without a theoretical framework.

As a result, the theoretical analysis indicates that research has progressively expanded from traditional consumer decision-making to technology adoption, communication, and user experience theories. Despite this evolution, the literature remains theoretically fragmented, with limited integration across disciplines. Future research should integrate consumer behavior, technology adoption, psychology, communication, and information systems theories to develop more comprehensive theoretical frameworks that explain the increasingly complex nature of consumer responses in relation to AI-driven marketing processes.

### 5.2 Context

This section provides a contextual perspective of prior studies to identify the situations in which they were conducted. Context refers to the circumstances under which research is conducted (Lim et al., 2021). Based on the literature review, the contexts were categorized into marketing communication, service marketing, online shopping and e-commerce, consumer behavior segments, and cultural settings, as presented in Table 4. The framework helps to explain how AI-driven marketing tactics affect consumption patterns in different contexts.

**Table 4:** Context framework.

Context	Sub-category	No. of articles
Marketing communication	Social media marketing	8
	Online advertising	6
	E-commerce marketing	31
Service marketing	Service failure management	2
	Banking and financial services	4
	Tourism services	5
	Community enterprise services	1
	E-commerce services	1
Online shopping and e-commerce	Food and grocery e-commerce	7
	Fashion and beauty e-commerce	5
	Modest fashion online shopping	1
	General retail e-commerce	9
Consumer behavior	Gen Z and youth consumers	2
	Female consumers	1
	Social media users	2
	Sustainable users/green consumer behavior	4
Healthcare, education, and entertainment	Healthcare marketing	3
	E-learning providers	1
	Music and entertainment industry marketing	1
	Charity advertising	1
Regions/countries	China	7
	Mauritius	1
	Indonesia	2
	Thailand	2
	India	3
	Sub-Saharan Africa	1
	Southeast Asia	1
	Mauritius	1

In the area of marketing communication, social media emerged as one of the most prominent contexts for examining consumer behavior. Studies highlighted the growing role of social media as a medium of communication between the firms and consumers (Hotkar et al., 2023; Jacobson et al., 2020; Amin, 2025). This indicates that AI-driven communication has transformed marketing from mass communication to hyper-personalized and interactive engagement. AI tactics enable marketers to deliver customized content, personalized recommendations, and targeted advertisements based on individual preferences, thereby enhancing consumer engagement and shaping behavioral responses. Online advertising was recognized as a significant communication channel through which brands influence consumer perceptions and decision-making (Lu and Chen, 2020; Lu et al., 2016 and Liu and Mattila, 2017). The increasing integration of AI into marketing demonstrates how automated emails and social media algorithms, that is, sentiment analysis, are reshaping consumer attention. The dominance of social media and e-commerce reflects the rapid transformation of marketing, in which AI technologies are used for personalization recommendations, targeted advertising, and consumer engagement. These AI tactics analyze large volumes of consumer data, and making them ideal environments for examining consumer reactions (Kasemrat et al., 2025). Consequently, they have received more scholarly attention than conventional offline marketing contexts.

Beyond communication, several studies examined AI-driven marketing tactics in digital commerce platforms, where marketing activities are integrated with transactional functions. Existing studies in e-commerce explored how recommendation systems, personalized content, and AI-enabled marketing tools influence online buying behavior and purchase decisions (Ma et al., 2024; Ding et al., 2025). This convergence of communication, recommendation systems and transactional capabilities highlights that AI-driven marketing tactics are no longer limited to promotions, but also embedded in digital commerce that influences consumers purchase decisions.

The studies highlighted the growing use of AI-driven tactics in service marketing contexts. A number of studies focused on service failure management and investigated how organizations use AI-driven marketing tactics to respond to service failures while maintaining customer satisfaction and loyalty (Cai et al., 2024; Zhou and Chang, 2024; Wang et al., 2024; Liu et al., 2023; Xing et al., 2022). These findings highlighted that AI-driven tactics not only support service delivery but also post-consumption services and long-term customer relationship management. Moreover, studies conducted in banking and financial services examined the role of service quality and consumer trust in shaping customer experiences (Li et al., 2023; Lee and Kim, 2023; Li and Zhang, 2023; Moran, 2024). Consumer engagement and service experiences were also widely explored in the tourism sector (Marti-Ochoa et al., 2025; Yan et al., 2025; Khrouf et al., 2025; Wang et al., 2024), while emerging contexts such as community enterprise services and e-commerce services require further investigation. Previous research has expanded across social media, e-commerce, tourism, banking, and service industries, and fewer studies examined AI-driven marketing tactics in healthcare, education, manufacturing, public services, and non-profit organizations. This reflects requirement of cross-sectoral research to better understand consumer behavior across diverse contextual. In contrast, non-commercial sectors have experienced comparatively less AI adoption and present greater organizational, ethical, and regulatory challenges (Taeihagh, 2021) which resulted limited empirical research. Future studies can extend AI-driven marketing research to these underexplored contexts to improve the generalizability.

### 5.3 Methodologies

Table 5 outlines the methods used in the previous studies. The findings indicate that a quantitative approach was predominantly used, particularly survey-based research dominated the literature (Bindah and Gunnoo, 2024; Hallikainen et al., 2022; Kim et al., 2023). Most prior studies used statistical techniques, such as PLS-SEM and SEM, to study the relationship among constructs. Some studies used qualitative methods, including interviews (Whitwam, 2025; Ding et al., 2025), to gain deeper insights of consumer experiences, while other researchers adopted advanced methods such as fuzzy-set qualitative comparative analysis (fsQCA) (Lalicic and Weismayer, 2021; Gupta et al., 2025). Furthermore, several systematic literature reviews and conceptual papers also contributed to knowledge development in the field (Jain et al., 2023; Adhikari and McFadden, 2025). However, the dominance of cross-sectional survey designs indicates a need for longitudinal, experimental and mixed-methods studies to understand consumer attitudes toward the AI-driven marketing tactics. Such approaches may offer new perspectives and contribute to the theoretical development in the domain.

**Table 5:** Methodology.

Method/ approach	Frequency	References
Survey-based method	51	Wang et al., 2024; Sadiq et al., 2025; Haneefa and Singh, 2025; Kumari et al., 2025; Bindah and Gunnoo, 2024; Kalogiannidis et al., 2024
Quantitative analytical studies	46	Huang, 2026; Xiu et al., 2025; Pantano and Pizzi, 2020; Yang et al., 2024; Abdalla et al., 2024; Yang et al., 2023
Experimental studies	26	Chen et al., 2025; Yan et al., 2025; Zhou and Chang, 2024; Lu and Zhang, 2025; Guo et al., 2023
Mixed-method studies	24	Pathak et al., 2025; Rui et al., 2025; Tran et al., 2021; Pantano et al., 2019
Conceptual/ literature studies	5	Adhikari and McFadden, 2025; Jain et al., 2023
Qualitative studies	3	Whitwam, 2025; Ding et al., 2025; Alyoubi, 2019

### 5.4 ADO framework

**Figure 6:**
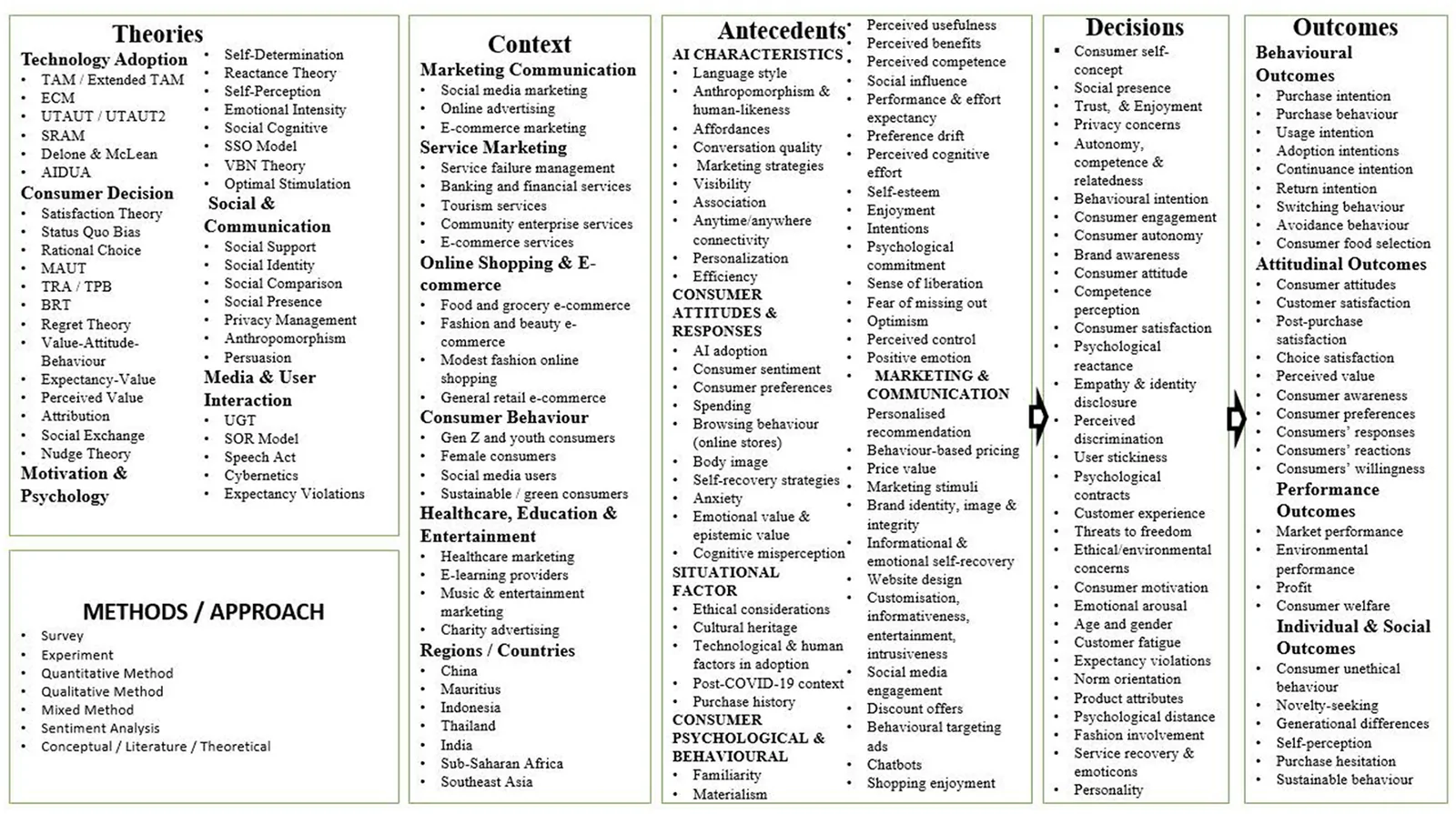
Comprehensive overview of literature on AI-driven marketing tactics and consumer behavior using TCM and ADO frameworks. Source: Adapted from Lim et al. (2021).

Understanding the interrelations among Antecedents–Decisions–Outcomes (ADO) is significant (Paul and Benito, 2018) for advancing theoretical and empirical research in this domain. To gain a deeper understanding of factors that shape consumer behavior in relation to AI-enabled marketing tactics, this study uses ADO framework by synthesizing previous studies and identifying patterns among the antecedents, decisions and outcomes. Figure 6 presents the ADO framework derived from the literature and highlights the structured relationships among these dimensions.

#### 5.4.1 Antecedents

Antecedents refer to causes, events and conditions that shape decisions and influence outcomes (Lim et al., 2021; Paul and Benito, 2018). This study develops an integrated antecedent framework that is classified into five categories: AI Characteristics, Consumer Psychological and Behavioral Traits, Consumer Attitudes and Responses, Marketing and Communication Factors, and Contextual/Situational Factors, as shown in Figure 6. The classification provides a structured understanding of how diverse drivers shape consumer behavior. The antecedents identified from previous studies were classified into five categories to provide a structured representation of the factors influencing consumer behavior.

This first category represents the technological characteristics of AI systems that directly influence consumer perceptions while interacting with AI-driven marketing tactics. It includes language style (task/social, fun/warm), anthropomorphism and human-likeness, transparency and authenticity, affordances, efficiency, and marketing strategies. These attributes influence consumer perceptions and interactions with AI, which subsequently affect customer satisfaction, ethical behavior, and consumer responses (Chen et al., 2025; Klein and Martinez, 2023; Li et al., 2024). The findings highlighted that AI communication features shape consumer perceptions and behavioral responses.

The second category comprises internal psychological and behavioral factors that shape how consumers perceive, evaluate, and respond to AI-driven marketing tactics. It includes factors such as familiarity, hedonic motivation, performance expectancy, effort expectancy, social influence, perceived benefit, and fear of missing out. These attributes highlight internal cognitive and motivational drivers that significantly shape consumer behavior (Arce-Urriza et al., 2025; Mahmoud et al., 2021). The dominance of these factors indicates that consumer behavior is influenced by both rational decisions and psychological motivations.

The third category captures consumers' affective reactions that emerge with exposure to AI marketing tactics. It includes factors, such as AI adoption, preferences, spending behavior, emotional value and epistemic value. These factors reflect evaluative and affective reactions toward AI-driven marketing stimuli (Gupta et al., 2025; Li et al., 2025). This highlights that consumer attitudes act as an important mechanism through which AI-driven marketing tactics influence behavioral outcomes.

The fourth category represents communication and promotional strategies, which were designed to influence consumer decision-making. It includes personalized recommendations, dynamic pricing, perceived price value, brand identity, website design and discount offers, targeted advertisements, chatbots, and shopping enjoyment.

These factors represent the communication mechanisms used to interact with consumers and influence their decision-making (Bindah and Gunnoo, 2024). The findings highlighted that AI-driven marketing tactics increasingly rely on personalized and interactive communication strategies to shape consumer's purchase behavior.

The fifth category represents external environmental and situational conditions that influence consumer behavior but remain outside marketers' direct control. It includes ethical considerations, cultural heritage, technological and human factors related to adoption, post-COVID-19, purchase history, and materialism. These factors provide the broader environmental and situational conditions under which consumers interact with AI-driven marketing tactics (Marti-Ochoa et al., 2025; Rui et al., 2025). The results highlight that consumer responses are not only determined by technological features and individual characteristics. Instead, they are shaped by circumstantial influences such as cultural values, ethical concerns, societal changes, and previous consumption experiences. This highlights the importance of considering situational conditions while examining the effectiveness of AI marketing tactics on consumer actions.

The integrative framework not only synthesizes existing literature but also highlights the interrelatedness of technological, psychological, and contextual drivers, providing a comprehensive foundation for future research to examine their combined and interactive effects. Moreover, the proposed antecedent framework highlights that AI-driven marketing tactics operate as stimuli that influence consumer responses through technological, psychological, and contextual mechanisms. This perspective extends prior reviews by shifting attention from AI as a technological innovation to AI-enabled marketing tactics as drivers of consumer decision-making. AI characteristics, marketing communication factors, and contextual conditions shape consumer perceptions, which also influence decision-making and behavioral outcomes. This perspective provides a structured overview of how AI tactics used by marketers influence consumer decisions beyond mere technology adoption.

#### 5.4.2 Decisions

Decisions are responses influenced by prior experiences, which lead to outcomes (Paul and Benito, 2018). In this domain, decisions include consumer's evaluations and behavioral intentions that emerge from the exposure of AI-driven marketing tactics. Figure 6, the conceptual map illustrates the relationship between antecedents and decisions identified from prior studies.

Within the decision framework, purchase intention is one of the most widely examined constructs, reflecting consumers' likelihood of being influenced by AI-driven marketing tactics. Several studies have shown that antecedents such as personalization, AI interaction and perceived usefulness influence purchase intention, primarily through mediating mechanisms. Furthermore, hedonic and utilitarian attitudes have been identified as mediators (Haneefa and Singh, 2025), which indicates that emotional and functional evaluations play a key role in decision-making.

Customer satisfaction is also one of the significant constructs examined in this domain. Prior studies highlighted that satisfaction is not solely influenced by AI marketing tactics but instead mediated by cognitive evaluations. Moreover, expectation violation has been identified as a key mediating variable through which chatbot interactions influence consumer satisfaction (Cai et al., 2024). This indicates that consumers constantly compare their actual experience with prior expectations. In a nutshell, the decision framework highlighted that consumer responses are shaped through cognitive and affective mechanisms. The finding also indicates that mediating processes play a crucial role in transforming technological, psychological, and marketing aspects into behavioral responses.

#### 5.4.3 Outcomes

Outcomes refer to the consequences of consumer perceptions and represent the final effects of AI-driven marketing tactics on consumer behavior (Paul and Benito, 2018). Previous studies predominantly examined outcomes such as consumer engagement, intention to use, purchase-related behavior, consumer satisfaction and service quality, which highlights their significance in evaluating the effectiveness of AI marketing tactics.

Among the recognized outcomes, customer engagement and purchase behavior are the most widely examined, which represent the effectiveness of AI-driven tactics in shaping customer experiences and strengthening customer relationships. Another frequent outcome is consumer's intention to use AI tools, which reflects their willingness to adopt AI technologies and their relevance in a digital environment. Purchase related outcomes are also a key outcome factor, with prior studies showing positive responses due to AI-driven interactions, primarily chatbots, that encourage favorable purchase behavior.

In a nutshell, the outcome framework indicates that AI-driven marketing tactics significantly influence consumer behavior and attitudes. The results highlighted that the effectiveness of AI marketing tactics depends not only on technical competencies but also on how consumers perceive, evaluate, and experience AI-driven interactions. The outcome factors may reflect either positive or negative consumer responses, which ultimately determine behavioral outcomes in the digital environment.

## 6 Future research directions

In the digital environment, artificial intelligence has become an essential component of modern marketing practices. This study synthesizes existing studies related to this field while identifying gaps in the literature. Based on this, several important directions for future research are proposed. The integration of bibliometric analysis and the TCM-ADO framework helps in identifying the research gap across theories, contexts, methodologies, antecedents, decisions, and outcomes, which collectively form the future research agenda.

### 6.1 Future directions: theory

This review indicates that a substantial proportion of prior research is based on traditional consumer decision-making theories and technology adoption theories, such as rational choice-based theories, TAM, and TPB. This theory framework assumes that consumers make logical and utility-maximizing decisions. Such assumptions may not capture the complexity of human behavior in an AI-driven environment, where decisions are often shaped by cognitive, emotional responses and algorithmic influences.

Future researchers can incorporate behavioral and psychological theories to explain consumers' decision-making based on cognitive and emotional responses to AI tactics used by brands. Drawing on behavioral economics, consumers are influenced by heuristics and algorithmic recommendations that influence their decisions. Similarly, there is a need to integrate psychological frameworks, such as self-determination theory and affordance theory, to better understand intrinsic motivation, perceived control and resistance toward AI-enabled tactics. Furthermore, there is a need to adopt interdisciplinary theoretical frameworks as existing literature applies theories in isolation. Future studies could integrate marketing, psychology, information systems, and human-computer interaction to develop comprehensive models. For instance, combining the stimulus-organism-response (SOR) model with technology adoption may offer a richer understanding of how AI-driven marketing tactics shape internal psychological states and behavioral outcomes. In addition, limited attention has been given to ethical and trust-based frameworks despite growing concerns of privacy, fairness and algorithmic bias. Future research can develop ethical AI frameworks that explain how transparency, fairness, accountability, and responsible AI tactics influence consumer trust and behavioral outcomes in this digital era.

### 6.2 Future directions: contexts

In terms of context (Figure 6), this review indicates that most studies focused on digital marketing communication and e-commerce, especially social media marketing, online advertising and retail platforms. However, fewer studies examined contexts such as fashion, community services, non-profit organizations and charity advertising. This concentration limits the generalizability of findings and highlights the need to examine AI-enabled marketing tactics in a wide range of industries. In the service sector, existing literature predominantly focuses on banking, tourism, and service failure management, while other emerging sectors such as public services, professional consultancy, entertainment, supply chain, and logistics remain underexplored. Future researchers may examine the influence of AI-driven tactics in diverse service sectors.

Furthermore, current research primarily focuses on Gen Z and active digital users, with less emphasis on older and less digitally engaged populations. In a same way, a substantial proportion of empirical research originates in India and China, while developing economies and cross-cultural studies remain underexplored.

### 6.3 Future directions: methodology

The methodologies section (Table 5) indicates that most of the prior studies used quantitative methods, such as survey and experimental research designs. However, these studies mainly collected cross-sectional data, which may limit the generalizability of the results in a dynamic world.

Conversely, a limited number of studies used qualitative methods, mixed methods and conceptual research, which indicates inadequate investigation of consumer experiences, perceptions and psychological mechanisms associated with AI-driven marketing tactics. Future researchers may incorporate interviews, focus groups, ethnography and case studies to gain deeper insights of consumer motivation and behavioral mechanisms.

In addition, fewer studies emphasize sentiment analysis and machine learning to analyze large volumes of data. Future studies can adopt longitudinal studies that provide deeper insights how consumer behavior evolves over time in response to AI tactics used by marketers. Furthermore, the integration of a qualitative and mixed-method approach can provide richer insights of consumer attitudes and experiences. Moreover, future studies may use supervised and unsupervised machine learning techniques to predict consumer experiences.

### 6.4 Future directions: antecedents

The analysis of the antecedents (Figure 6) showed that prior studies focused on AI characteristics such as personalization, anthropomorphism, and efficiency as well as marketing and communication factors including recommendations, pricing and social media engagement. Consumer psychological traits factors include perceived usefulness, social influence and fear of missing out; these factors help in understanding consumer decisions in the context of AI marketing tactics. However, several potential antecedents remain underexplored.

Future research can extend this domain by examining psychological and situational factors such as perceived control, emotional value, and consumer sense of liberation. In addition, future studies can further examine contextual antecedents including ethical concerns, cultural heritage, and post-pandemic behavioral changes to better understand their influence on consumer decision-making.

Moreover, future studies may analyze interaction effects and moderation effects such as the interaction between personalization and privacy concerns. In future, there is also a need to examine evolving AI capabilities, such as chatbot communication quality, generative AI content and algorithm pricing. In addition, researchers may investigate emerging behavioral patterns, including digital anxiety, information overload, self-recovery strategies, and browsing behavior, to better understand the complex mechanisms.

### 6.5 Future directions: decisions

This review shows that previous studies mainly focused on behavioral intention, consumer attitude and satisfaction as decision variables. Although, these constructs provide valuable insights into consumer decision-making processes, they offer a relatively limited perspective on the broader psychological and behavioral consequences associated with AI-driven marketing interactions. However, factors such as psychological reactance, perceived discrimination, user stickiness, customer fatigue and autonomy are significant emerging factors that remain underexplored despite their increasing relevance in the digital environment.

Moreover, it is necessary to recognize that consumer actions in the context of AI marketing tactics are not only rational but also shaped by emotional, cognitive and situational factors. Therefore, future researchers can focus on empathy, social presence, emotional arousal and self-concept as these factors may provide a deeper understanding of how consumers respond to AI-driven marketing tactics. In addition, AI characteristics, including anthropomorphism, personalization, and language style, influence behavioral outcomes such as switching intention, loyalty, and sustainable behavior.

### 6.6 Future directions: outcomes

The relationship map (Figure 6) indicates that existing studies primarily examined purchase intention, adoption intention and purchase behavior. While these outcomes remain important indicators of marketing effectiveness, relatively few studies explored attitudinal outcomes, social outcomes and long-term consequences associated with AI marketing tactics. Future research may expand the scope of outcomes by focusing on post-purchase satisfaction, consumer awareness and preferences. Future studies may focus on organizational outcomes such as market performance, profitability, environmental performance, and consumer welfare to provide a wide-ranging assessment of AI marketing tactics. The review highlights that existing studies mainly focused on factors such as self-perception, generational differences, consumer unethical behavior and sustainable behaviors. Future research may examine the influence of AI characteristics, personalization strategies, chatbots, targeted advertising, and social media engagement by considering both individual and societal-level factors, which help in understanding the implications of AI marketing tactics in contemporary digital environments. Moreover, future studies should understand how cognitive, emotional and psychological mechanisms mediate the relationship between the AI-driven marketing tactics and consumer behavioral outcomes.

## 7 Conclusion

This study used a hybrid review approach with bibliometric analysis, SPAR-4-SLR, and the TCM-ADO framework to provide a comprehensive synthesis of the literature on AI-driven marketing tactics and consumer behavior. The finding reveals substantial growth of AI-driven marketing tactics and synthesizes previous studies on how these tactics influence consumers' cognitive, emotional, and behavioral decision-making processes. Unlike previous reviews that examined artificial intelligence as a broad technology, this study focuses on AI-driven marketing tactics and provides a structured synthesis of how these tactics influence consumers cognitive, emotional, and behavioral responses.

The review also highlighted that previous studies emphasized consumer decision-making and technology adoption theories such as TAM and UTAUT. While these theories explain acceptance and usage intention of AI, they provide limited understanding of emotions, ethics and psychological consequences. The study identifies underexplored research areas such as consumer fatigue, privacy concerns, perceived discrimination, sustainable behavior, and unethical consumer behavior. Moreover, few previous studies explored cross-cultural and longitudinal investigations to examine the effects of AI personalization and automation.

From a practical perspective, the finding provides implications for marketers. Businesses increasingly use AI-driven tactics such as personalization, chatbots, targeted advertising, sentiment analysis, automated emails, AI-driven pricing, and AI-curated content to enhance consumer engagement. However, AI personalization may raise ethical concerns, privacy risks, and perceptions of reduced consumer autonomy (Waqas and Qadri, 2025). Therefore, organizations should focus on transparency, fairness, and ethical AI practices to influence consumer engagement and long-term relationships.

This review has certain limitations. First, this article includes only Scopus-indexed journals and excludes relevant studies indexed in other databases. Secondly, this study focuses on AI-driven marketing tactics and consumer behavior rather than AI as a broader technological phenomenon. As a consequence, studies examining AI as a broad, which resulted outside the marketing context and were beyond the scope of the review. Thirdly, this study includes only English language articles published between 2016 and 2026, which may have excluded relevant studies published in other languages.

In a nutshell, this study synthesizes the existing studies on how AI-driven marketing influences consumer behavior through cognitive, emotional and behavioral mechanisms. The integration of bibliometric analysis with SPAR-4-SLR protocol and the TCM-ADO framework offers a structured understanding of future theoretical, methodological, and practical research on AI-driven marketing tactics.

## References

[B1] Abdalla H. B. Gheisari M. Awlla A. H. (2024). Hybrid self-attention BiLSTM and incentive learning-based collaborative filtering for e-commerce recommendation systems. Electr. Commer. Res. 25, 1–24. doi: 10.2139/ssrn.5239678

[B2] Adhikari S. McFadden B. R. (2025). Bridging taste and health: the role of machine learning in consumer food selection. Int. Food Agribus. Manage. Rev. 1, 1–16. doi: 10.22434/ifamr1131

[B3] Ahmad S. A. Mir M. A. (2025). “Impact of artificial Intelligence on marketing and consumer decision-making,” in Generative Artificial Intelligence and Ethics: Standards, Guidelines, and Best Practices, ed. L. Gaur (Hershey, PA: IGI Global), 169–188. doi: 10.4018/979-8-3693-3691-5.ch008

[B4] Alyoubi B. A. (2019). The impact of big data on electronic commerce in profit organisations in Saudi Arabia. Res. World Econ. 10, 106–115. doi: 10.5430/rwe.v10n4p106

[B5] Al-Zubidy A. Carver J. C. (2019). Identification and prioritization of SLR search tool requirements: an SLR and a survey. Empir. Softw. Eng. 24, 139–169. doi: 10.1007/s10664-018-9626-5

[B6] Amin A. (2025). Artificial intelligence in social media: a catalyst for impulse buying behavior? Young Consum. 26, 765–785. doi: 10.1108/YC-10-2024-2297

[B7] Arce-Urriza M. Chocarro R. Cortinas M. Marcos-Matas G. (2025). From familiarity to acceptance: the impact of generative artificial intelligence on consumer adoption of retail chatbots. J. Retail. Consum. Serv. 84:104234. doi: 10.1016/j.jretconser.2025.104234

[B8] Arenas-Gaitán J. Sanz-Altamira B. Ramírez-Correa P. E. (2019). Complexity of understanding consumer behavior from the marketing perspective. Complexity 2019, 1–3. doi: 10.1155/2019/2837938

[B9] AWS (Amazon Personalize) (2021). Amazon personalize: create real-time personalized user experiences faster at scale. Amazon. Available online at: https://aws.Amazon.com/personalize/ (Accessed April 30, 2026).

[B10] Bag S. Srivastava G. Bashir M. M. A. Kumari S. Giannakis M. Chowdhury A. H. . (2022). Journey of customers in this digital era: understanding the role of artificial intelligence technologies in user engagement and conversion. Benchmarking 29, 2074–2098. doi: 10.1108/BIJ-07-2021-0415

[B11] Batham S. Arora H. Gupta V. (2025). Investigation of the antecedents of personal saving behavior: a systematic literature review using TCM-ADO framework. J. Risk Financ. Manage. 18:554. doi: 10.3390/jrfm18100554

[B12] Bazavan A. Huidumac-Petrescu C. E. (2022). “China's drive for technological leadership in artificial intelligence. Key policies and government-industry integration,” in International conference on business excellence, ed. M. Busu (Cham: Springer Nature Switzerland), 17–30. doi: 10.1007/978-3-031-19886-1_2

[B13] Berg A. (2026). The impact of AI-personalised algorithms on consumers' purchase decisions and impulsive purchasing (Thesis). HAMK University of Applied Sciences.

[B14] Bindah E. Gunnoo L. (2024). Consumer behaviour towards sponsored-labelled targeted advertisements on Meta platforms in the context of Mauritius. J. Bus. Econ. Manage. 25, 175–190. doi: 10.3846/jbem.2024.21109

[B15] Bohara S. Gupta G. Kumar V. (2025). Integrating artificial intelligence and consumer behavior: insights and directions from a hybrid systematic literature review. J. Mark. Commun. 1–30. doi: 10.1080/13527266.2025.2508181

[B16] Cai N. Gao S. Yan J. (2024). How the communication style of chatbots influences consumers' satisfaction, trust, and engagement in the context of service failure. Humanit. Soc. Sci. Commun. 11, 1–11. doi: 10.1057/s41599-024-03212-0

[B17] Chen J. Ke W. Wu Y. Zhang J. (2025). Do cute language style chatbots make consumers more unethical? Int. J. Inf. Manage. 85:102941. doi: 10.1016/j.ijinfomgt.2025.102941

[B18] Chiu W. T. Ho Y. S. (2007). Bibliometric analysis of tsunami research. Scientometrics 73, 3–17. doi: 10.1007/s11192-005-1523-132214553

[B19] Danese P. Manfè V. Romano P. (2018). A systematic literature review on recent lean research: state-of-the-art and future directions. Int. J. Manage. Rev. 20, 579–605. doi: 10.1111/ijmr.12156

[B20] Davenport T. Guha A. Grewal D. Bressgott T. (2020). How artificial intelligence will change the future of marketing. J. Acad. Mark. Sci. 48, 24–42. doi: 10.1007/s11747-019-00696-0

[B21] De Bruyn A. Viswanathan V. Beh Y. S. Brock J. K.-U. Von Wangenheim F. (2020). Artificial intelligence and marketing: pitfalls and opportunities. J. Interact. Mark. 51, 91–105. doi: 10.1016/j.intmar.2020.04.007

[B22] Ding L. Antonucci G. Venditti M. (2025). Unveiling user responses to AI-powered personalised recommendations: a qualitative study of consumer engagement dynamics on Douyin. Qual. Mark. Res. 28, 234–255. doi: 10.1108/QMR-11-2023-0151

[B23] Fahimnia B. Sarkis J. Davarzani H. (2015). Green supply chain management: a review and bibliometric analysis. Int. J. Prod. Econ. 162, 101–114. doi: 10.1016/j.ijpe.2015.01.003

[B24] Gao B. Wang Y. Xie H. Hu Y. Hu Y. (2023). Artificial intelligence in advertising: advancements, challenges, and ethical considerations in targeting, personalization, content creation, and ad optimization. Sage Open 13:21582440231210759. doi: 10.1177/21582440231210759

[B25] Guo W. Tian J. Li M. (2023). Price-aware enhanced dynamic recommendation based on deep learning. J. Retail. Consum. Serv. 75:103500. doi: 10.1016/j.jretconser.2023.103500

[B26] Gupta S. Arora A. Singh S. Jain J. (2025). AI feel millennials: prioritizing the intentions towards adoption of AI-enabled chatbots using fuzzy-AHP approach. J. Sci. Technol. Policy Manage. doi: 10.1108/JSTPM-09-2023-0159. [Epub ahead of print].

[B27] Haleem A. Javaid M. Qadri M. A. Singh R. P. Suman R. (2022). Artificial intelligence (AI) applications for marketing: a literature-based study. Int. J. Intell. Netw. 3, 119–132. doi: 10.1016/j.ijin.2022.08.005

[B28] Hallikainen H. Luongo M. Dhir A. Laukkanen T. (2022). Consequences of personalized product recommendations and price promotions in online grocery shopping. J. Retail. Consum. Serv. 69:103088. doi: 10.1016/j.jretconser.2022.103088

[B29] Haneefa K. V. Singh H. (2025). Does AI matter in marketing? Unveiling the role of AI-driven marketing in quick grocery. Int. Rev. Retail Distrib. Consum. Res. 36, 1–23. doi: 10.1080/09593969.2025.2526482

[B30] Hendrayati H. Achyarsyah M. Marimon F. Hartono U. Putit L. (2024). The impact of artificial intelligence on digital marketing: leveraging potential in a competitive business landscape. Emerg. Sci. J. 8, 2343–2359. doi: 10.28991/ESJ-2024-08-06-012

[B31] Hossain M. S. (2024). Textual feature engineering for purchase intent and customer satisfaction: insights from marketing 4.0 and sentiment. Sustain. Futures 8:100385. doi: 10.1016/j.sftr.2024.100385

[B32] Hotkar P. Garg R. Sussman K. (2023). Strategic social media marketing: an empirical analysis of sequential advertising. Prod. Oper. Manage. 32, 4005–4020. doi: 10.1111/poms.14075

[B33] Huang D. Markovitch D. G. Stough R. A. (2024). Can chatbot customer service match human service agents on customer satisfaction? An investigation in the role of trust. J. Retail. Consum. Serv. 76:103600. doi: 10.1016/j.jretconser.2023.103600

[B34] Huang S. Y. (2026). Asking fintech voice chatbots: explaining consumer switching intention by status quo bias theory. J. Retail. Consum. Serv. 88:104457. doi: 10.1016/j.jretconser.2025.104457

[B35] Jacobson J. Gruzd A. Hernández-García Á. (2020). Social media marketing: who is watching the watchers? J. Retail. Consum. Serv. 53:101774. doi: 10.1016/j.jretconser.2019.03.001

[B36] Jain V. Wadhwani K. Eastman J. K. (2023). Artificial intelligence consumer behaviour: a hybrid review and research agenda. J. Consum. Behav. 23, 676–697. doi: 10.1002/cb.2233

[B37] Jaziri D. Rehman M. A. Hasni M. J. S. Ben Jebril A. (2025). Food anti-consumption behavior: a systematic review and future research agenda. Br. Food J. 127, 373–395. doi: 10.1108/BFJ-10-2024-1087

[B38] Kalogiannidis S. Patitsa C. Chalaris M. (2024). The integration of artificial intelligence in business communication channels: opportunities and challenges. WSEAS Trans. Bus. Econ. 21, 1922–1944. doi: 10.37394/23207.2024.21.157

[B39] Kasemrat R. Kraiwanit T. Khaothawirat A. Chinnapha S. Luo Q. (2025). Benchmarking machine learning models for predictive analytics in e-commerce strategy. Corp. Bus. Strategy Rev. 6, 146–155.

[B40] Khrouf L. Azzabi M. Bouchnak M. (2025). Predicting continuance intention to use chatbots in tourism commercial websites. J. Hosp. Tour. Insights 8, 3687–3704. doi: 10.1108/JHTI-01-2025-0125

[B41] Kim J. (2025). Advertising in the age of agentic AI: call for research. J. Interact. Advert. 25, 215–221. doi: 10.1080/15252019.2025.2557107

[B42] Kim J. Y. Li Y. Choi J. (2023). Understanding the continuance intention to use chatbot services. Asia Mark. J. 25:1. doi: 10.53728/2765-6500.1613

[B43] Kim K. Park O. J. Yun S. Yun H. (2017). What makes tourists feel negatively about tourism destinations? Application of hybrid text mining methodology to smart destination management. Technol. Forecast. Soc. Change 123, 362–369. doi: 10.1016/j.techfore.2017.01.001

[B44] Klein K. Martinez L. F. (2023). The impact of anthropomorphism on customer satisfaction in chatbot commerce: an experimental study in the food sector. Electr. Commer. Res. 23, 2789–2825. doi: 10.1007/s10660-022-09562-8

[B45] Kopalle P. K. Pauwels K. Akella L. Y. Gangwar M. (2023). Dynamic pricing: definition, implications for managers, and future research directions. J. Retail. 99, 580–593. doi: 10.1016/j.jretai.2023.11.003

[B46] Kumar A. Bezawada R. Rishika R. Janakiraman R. Kannan P. K. (2016). From social to sale: the effects of firm-generated content in social media on customer behavior. J. Mark. 80, 7–25. doi: 10.1509/jm.14.0249

[B47] Kumari P. Gupta N. Aeron P. (2025). The rise of the phygital citizens: generative AI experiences and customer citizenship behaviour. J. Glob. Inf. Manage. 33, 1–17. doi: 10.4018/JGIM.373320

[B48] Lalicic L. Weismayer C. (2021). Consumers' reasons and perceived value co-creation of using artificial intelligence-enabled travel service agents. J. Bus. Res. 129, 891–901. doi: 10.1016/j.jbusres.2020.11.005

[B49] Lee M. Kim H. J. (2023). A collaborative filtering model incorporating media promotions and users' variety-seeking tendencies in the digital music market. Decis. Support Syst. 174:114022. doi: 10.1016/j.dss.2023.114022

[B50] Li B. Yao R. Nan Y. (2024). How does anthropomorphism promote consumer responses to social chatbots: mind perception perspective. Internet Res. 35, 2471–2495. doi: 10.1108/INTR-04-2024-0583

[B51] Li C. Y. Fang Y. H. Chiang Y. H. (2023). Can AI chatbots help retain customers? An integrative perspective using affordance theory and service-domain logic. Technol. Forecast. Soc. Change 197:122921. doi: 10.1016/j.techfore.2023.122921

[B52] Li C. Y. Zhang J. T. (2023). Chatbots or me? Consumers' switching between human agents and conversational agents. J. Retail. Consum. Serv. 72:103264. doi: 10.1016/j.jretconser.2023.103264

[B53] Li T. Wu Y. Liu Y. Li J. (2025). Deep learning-based analysis of e-commerce enterprises: user behavior and consumption prediction. J. Organ. End User Comput. 37, 1–36. doi: 10.4018/JOEUC.379722

[B54] Lim W. M. Kumar S. (2024). Guidelines for interpreting the results of bibliometrics analysis: a sensemaking approach. Glob. Bus. Organ. Excell. 43, 17–26. doi: 10.1002/joe.22229

[B55] Lim W. M. Yap S. F. Makkar M. (2021). Home sharing in marketing and tourism at a tipping point: what do we know, how do we know, and where should we be heading?. J. Bus. Res. 122, 534–566. doi: 10.1016/j.jbusres.2020.08.051 33012896 PMC7523531

[B56] Liu D. Lv Y. Huang W. (2023). How do consumers react to chatbots' humorous emojis in service failures. Technol. Soc. 73:102244. doi: 10.1016/j.techsoc.2023.102244

[B57] Liu S. Q. Mattila A. S. (2017). Airbnb: online targeted advertising, sense of power, and consumer decisions. Int. J. Hosp. Manage. 60, 33–41. doi: 10.1016/j.ijhm.2016.09.012

[B58] Lu X. Chen Y. (2020). Situations matter: understanding how individual browsing situation routineness impacts online users' advertisement clicks behavior. J. Electr. Commer. Res. 21, 113–129.

[B59] Lu X. Zhao X. Xue L. (2016). Is combining contextual and behavioral targeting strategies effective in online advertising? ACM Trans. Manage. Inf. Syst. 7, 1–20. doi: 10.1145/2883816

[B60] Lu Y. Zhang J. (2025). Balancing identity diversity and product contexts: understanding consumer trust in AI-enhanced chatbot services. J. Retail. Consum. Serv. 84:104205. doi: 10.1016/j.jretconser.2024.104205

[B61] Ma X. Li Y. Asif M. (2024). E-commerce review sentiment analysis and purchase intention prediction based on deep learning technology. J. Organ. End User Comput. 36, 1–29. doi: 10.4018/JOEUC.335122

[B62] Ma X. Wang P. (2024). An in-depth analysis and prediction study of consumer buying behaviour for digital marketing. Appl. Math. Nonlinear Sci. 9:20242814. doi: 10.2478/amns-2024-2814

[B63] Mahmoud A. B. Hack-Polay D. Grigoriou N. Mohr I. Fuxman L. (2021). A generational investigation and sentiment and emotion analyses of female fashion brand users on Instagram in Sub-Saharan Africa. J. Brand Manage. 28:526. doi: 10.1057/s41262-021-00244-8

[B64] Majcher S. (2025). Consumers' perspective of plant-based meat alternatives—a systematic literature review and future research agenda. Int. J. Consum. Stud. 49:e70036. doi: 10.1111/ijcs.70036

[B65] Malthouse E. C. Hessary Y. K. Vakeel K. A. Burke R. Fudurić M. (2019). An algorithm for allocating sponsored recommendations and content: unifying programmatic advertising and recommender systems. J. Advert. 48, 366–379. doi: 10.1080/00913367.2019.1652123

[B66] Mariani M. Borghi M. (2019). Industry 4.0: a bibliometric review of its managerial intellectual structure and potential evolution in the service industries. Technol. Forecast. Soc. Change 149:119752. doi: 10.1016/j.techfore.2019.119752

[B67] Mariani M. M. Perez-Vega R. Wirtz J. (2022). AI in marketing, consumer research and psychology: a systematic literature review and research agenda. Psychol. Mark. 39, 755–776. doi: 10.1002/mar.21619

[B68] Marti-Ochoa J. Martin-Fuentes E. Ferrer-Rosell B. (2025). AI-driven virtual travel influencers and ethical consumerism: analysing engagement with Sena Zaro's Instagram content. Young Consum. 26, 728–743. doi: 10.1108/YC-08-2024-2204

[B69] Moran N. (2024). Losing privacy versus losing choice: how consumers react to different costs of personalization. J. Consum. Aff. 58, 587–605. doi: 10.1111/joca.12584

[B70] Nanda A. Xu Y. Zhang F. (2021). How would the COVID-19 pandemic reshape retail real estate and high streets through acceleration of E-commerce and digitalization? J. Urban Manage. 10, 110–124. doi: 10.1016/j.jum.2021.04.001

[B71] Nirmal D. D. Nageswara Reddy K. Singh S. K. (2024). Application of fuzzy methods in green and sustainable supply chains: critical insights from a systematic review and bibliometric analysis. Benchmarking 31, 1700–1748. doi: 10.1108/BIJ-09-2022-0563

[B72] Pantano E. Giglio S. Dennis C. (2019). Making sense of consumers' tweets: sentiment outcomes for fast fashion retailers through Big Data analytics. Int. J. Retail Distrib. Manage. 47, 915–927. doi: 10.1108/IJRDM-07-2018-0127

[B73] Pantano E. Pizzi G. (2020). Forecasting artificial intelligence on online customer assistance: evidence from chatbot patents analysis. J. Retail. Consum. Serv. 55:102096. doi: 10.1016/j.jretconser.2020.102096

[B74] Pathak K. Prakash G. Samadhiya A. Kumar A. Luthra S. (2025). Impact of Gen-AI chatbots on consumer services experiences and behaviors: focusing on the sensation of awe and usage intentions through a cybernetic lens. J. Retail. Consum. Serv. 82:104120. doi: 10.1016/j.jretconser.2024.104120

[B75] Patil R. Shivashankar K. Porapur S. M. Kagawade S. (2024). The role of AI-driven social media marketing in shaping consumer purchasing behaviour: an empirical analysis of personalization, predictive analytics, and engagement. ITM Web Conf. 68:01032. doi: 10.1051/itmconf/20246801032

[B76] Paul J. Benito G. R. (2018). A review of research on outward foreign direct investment from emerging countries, including China: what do we know, how do we know and where should we be heading? Asia Pac. Bus. Rev. 24, 90–115. doi: 10.1080/13602381.2017.1357316

[B77] Paul J. Criado A. R. (2020). The art of writing literature review: what do we know and what do we need to know? Int. Bus. Rev. 29:101717. doi: 10.1016/j.ibusrev.2020.101717

[B78] Paul J. Feliciano-Cestero M. M. (2021). Five decades of research on foreign direct investment by MNEs: an overview and research agenda. J. Bus. Res. 124, 800–812. doi: 10.1016/j.jbusres.2020.04.017 32292218 PMC7151309

[B79] Paul J. Lim W. M. O'Cass A. Hao A. W. Bresciani S. (2021). Scientific procedures and rationales for systematic literature reviews (SPAR-4-SLR). Int. J. Consum. Stud. 45, O1–O16. doi: 10.1111/ijcs.12695

[B80] Paul J. Parthasarathy S. Gupta P. (2017). Exporting challenges of SMEs: a review and future research agenda. J. World Bus. 52, 327–342. doi: 10.1016/j.jwb.2017.01.003

[B81] Phulwani P. R. Kumar D. Goyal P. (2020). A systematic literature review and bibliometric analysis of recycling behavior. J. Glob. Mark. 33, 354–376. doi: 10.1080/08911762.2020.1765444

[B82] Pizzi G. Scarpi D. Pantano E. (2021). Artificial intelligence and the new forms of interaction: who has the control when interacting with a chatbot? J. Bus. Res. 129, 878–890. doi: 10.1016/j.jbusres.2020.11.006

[B83] Pranckut R. (2021). Web of science (WoS) and scopus: the titans of bibliographic information in today's academic world. Publications 9, 1–59. doi: 10.3390/publications9010012

[B84] Puntoni S. Reczek R. W. Giesler M. Botti S. (2021). Consumers and artificial intelligence: an experiential perspective. J. Mark. 85, 131–151. doi: 10.1177/0022242920953847

[B85] Rui M. Sparacino A. Merlino V. M. Brun F. Massaglia S. Blanc S. . (2025). Exploring consumer sentiments and opinions in wine E-commerce: a cross-country comparative study. J. Retail. Consum. Serv. 82:104097. doi: 10.1016/j.jretconser.2024.104097

[B86] Sadiq S. Kaiwei J. Aman I. Mansab M. (2025). Examine the factors influencing the behavioral intention to use social commerce adoption and the role of AI in SC adoption. Eur. Res. Manage. Bus. Econ. 31:100268. doi: 10.1016/j.iedeen.2024.100268

[B87] Sahoo S. (2022). Big data analytics in manufacturing: a bibliometric analysis of research in the field of business management. Int. J. Prod. Res. 60, 6793–6821. doi: 10.1080/00207543.2021.1919333

[B88] Santos S. Gonçalves H. M. Teles M. (2023). Social media engagement and real-time marketing: using net-effects and set-theoretic approaches to understand audience and content-related effects. Psychol. Mark. 40, 497–515. doi: 10.1002/mar.21756

[B89] Senyapar H. N. D. (2024). Artificial intelligence in marketing communication: a comprehensive exploration of the integration and impact of AI. Technium Soc. Sci. J. 55:64. doi: 10.47577/tssj.v55i1.10651

[B90] Shi Q. Shen L. (2025). Advancing firm-level digital technology diffusion: a hybrid bibliometric and framework-based systematic literature review. Systems 13:262. doi: 10.3390/systems13040262

[B91] Singh A. K. Negi D. (2025). Save today, secure tomorrow: a systematic review of literature on retirement planning behaviour using the TCM-ADO framework. Emerald. doi: 10.1108/S2754-58652025000004B005

[B92] Song M. Xing X. Duan Y. Cohen J. Mou J. (2022). Will artificial intelligence replace human customer service? The impact of communication quality and privacy risks on adoption intention. J. Retail. Consum. Serv. 66:102900. doi: 10.1016/j.jretconser.2021.102900

[B93] Sterne J. (2017). Artificial Intelligence for Marketing: Practical Applications. Hoboken, NJ: John Wiley & Sons, Inc.

[B94] Summers C. A. Smith R. W. Reczek R. W. (2016). An audience of one: behaviorally targeted ads as implied social labels. J. Consum. Res. 43, 156–178. doi: 10.1093/jcr/ucw012

[B95] Taeihagh A. (2021). Governance of artificial intelligence. Policy Soc. 40, 137–157. doi: 10.1080/14494035.2021.1928377

[B96] Tran A. D. Pallant J. I. Johnson L. W. (2021). Exploring the impact of chatbots on consumer sentiment and expectations in retail. J. Retail. Consum. Serv. 63:102718. doi: 10.1016/j.jretconser.2021.102718

[B97] Verhoef P. C. Broekhuizen T. Bart Y. Bhattacharya A. Qi Dong J. Fabian N. . (2021). Digital trans formation: a multidisciplinary reflection and research agenda. J. Bus. Res. 122, 889–901. doi: 10.1016/j.jbusres.2019.09.022

[B98] Villarroel Ordenes F. Ludwig S. De Ruyter K. Grewal D. Wetzels M. (2017). Unveiling what is written in the stars: analyzing explicit, implicit, and discourse patterns of sentiment in social media. J. Consum. Res. 43, 875–894. doi: 10.1093/jcr/ucw070

[B99] Wang L. Xiao J. Luo Z. Guo Y. Xu X. A. (2024). The impact of default options on tourist intention post tourism chatbot failure: the role of service recovery and emoticon. Tour. Manage. Perspect. 53:101299. doi: 10.1016/j.tmp.2024.101299

[B100] Waqas M. Qadri U. A. (2025). When Consumer autonomy is compromised: adopting a dual-type view to understand AI-driven marketing and its impact on young consumers' ethical behavior. J. Bus. Ethics, 1–19. doi: 10.1007/s10551-025-06155-x

[B101] Whitwam B. (2025). Global cultural convergence in social commerce: how platforms transform trust-building among Gen Z Consumers. J. Internet Commer. 24, 1–23. doi: 10.1080/15332861.2025.2547067

[B102] Xing X. Song M. Duan Y. Mou J. (2022). Effects of different service failure types and recovery strategies on the consumer response mechanism of chatbots. Technol. Soc. 70:102049. doi: 10.1016/j.techsoc.2022.102049

[B103] Xiu T. Sun Y. Zhang X. Gao Y. Wu J. Zhang A. Y. . (2025). The analysis of emotion-aware personalized recommendations via multimodal data fusion in the field of art. J. Organ. End User Comput. 37, 1–29. doi: 10.4018/JOEUC.368008

[B104] Yan H. Tian T. Xiong H. (2025). The power of emojis: enhancing the willingness to adopt chatbot recommendations. J. Retail. Consum. Serv. 87:104388. doi: 10.1016/j.jretconser.2025.104388

[B105] Yang Q. Zhu B. Liao H. Wu X. (2023). Learning consumer preferences from online textual reviews and ratings based on the aggregation-disaggregation paradigm with attitudinal Choquet integral. Econ. Res. 36:2106282. doi: 10.1080/1331677X.2022.2106282

[B106] Yang Z. Li Q. Islam N. Han C. Gupta S. (2024). Product attribute and heterogeneous sentiment analysis-based evaluation to support online personalized consumption decisions. IEEE Trans. Eng. Manage. 71, 11198–11211. doi: 10.1109/TEM.2024.3413999

[B107] Zhao L. Fu B. Bai S. (2025). Understanding the influence of personalized recommendation on purchase intentions from a self-determination perspective: contingent upon product categories. J. Theor. Appl. Electr. Commer. Res. 20:32. doi: 10.3390/jtaer20010032

[B108] Zhou C. Chang Q. (2024). Informational or emotional? Exploring the relative effects of chatbots' self-recovery strategies on consumer satisfaction. J. Retail. Consum. Serv. 78:103779. doi: 10.1016/j.jretconser.2024.103779

[B109] Zhu J. Liu W. (2020). A tale of two databases: the use of Web of Science and Scopus in academic papers. Scientometrics 123, 321–335. doi: 10.1007/s11192-020-03387-8

[B110] Ziakis C. Vlachopoulou M. (2023). Artificial intelligence in digital marketing: insights from a comprehensive review. Information 14:664. doi: 10.3390/info14120664

[B111] Zupic I. Cater T. (2015). Bibliometric methods in management and organization. Organ. Res. Methods 18, 429–472. doi: 10.1177/1094428114562629

